# Time-Dependent Effect of Seepage Force on Initiation of Hydraulic Fracture around a Vertical Wellbore

**DOI:** 10.3390/ma16052012

**Published:** 2023-02-28

**Authors:** Hyonchol Rim, Youliang Chen, Jun Tokgo, Xi Du, Yi Li, Suran Wang

**Affiliations:** 1 Department of Civil Engineering, School of Environment and Architecture, University of Shanghai for Science and Technology, Shanghai 200093, China; 2 College of Architecture and Urban Planning, Tongji University, Shanghai 200092, China

**Keywords:** stress field, unsteady seepage, seepage force, pore pressure, fracture initiation

## Abstract

Fluid penetration into the rock during hydraulic fracturing has been an essential issue in studying the mechanism of fracture initiation, especially the seepage force caused by fluid penetration, which has an important effect on the fracture initiation mechanism around a wellbore. However, in previous studies, the effect of seepage force under unsteady seepage on the fracture initiation mechanism was not considered. In this study, a new seepage model that can predict the variations of pore pressure and seepage force with time around a vertical wellbore for hydraulic fracturing was established by using the method of separation of variables and the Bessel function theory. Then, based on the proposed seepage model, a new circumferential stress calculation model considering the time-dependent effect of seepage force was established. The accuracy and applicability of the seepage model and the mechanical model were verified by comparison with numerical, analytical and experimental results. The time-dependent effect of seepage force on fracture initiation under unsteady seepage was analyzed and discussed. The results show that when the wellbore pressure is constant, the circumferential stress induced by seepage force increases over time, and the possibility of fracture initiation also increases. The higher the hydraulic conductivity, the lower the fluid viscosity and the shorter the time required for tensile failure during hydraulic fracturing. In particular, when the tensile strength of rock is lower, the fracture initiation may occur within the rock mass rather than on the wellbore wall. This study is promising to provide a theoretical basis and practical guidance for further research on fracture initiation in the future.

## 1. Introduction

The hydraulic fracturing technique has been widely applied in the field of rock engineering practice, such as hydrocarbon extraction, geothermal, mining and other industries to enhance production by increasing the permeability of the rock matrix [1,2,3,4]. Hydraulic fracturing is a mechanical process of injecting fluid into a wellbore, which is closely related to the seepage field and stress field around the wellbore [5,6,7,8,9]. Therefore, the accurate evaluation of the seepage field and stress field around the wellbore is an essential issue in studying the hydraulic fracturing mechanism. In particular, with various fluids (such as SC-CO_2_ fluid) beneficial for environmental protection, the influence of the seepage field on fracture initiation is becoming increasingly apparent [10,11,12,13,14].

The prediction of hydraulic fracture initiation pressure in a wellbore is one of the foundations for hydraulic fracture design and in situ stress measurement [15]. The initial study of hydraulic fracturing started from kirsch’s solution, which is an analytical solution for the stress field in impermeable reservoir rocks. Afterwards, the stress solution for permeable rocks considering poroelastic stress was derived by Haimson and Fairhurst (1967) [16]. These two stress solutions have played an important role in the study of fracture initiation mechanisms during hydraulic fracturing. Based on their research, many scholars developed different models that can predict the fracture initiation pressure. Table 1 lists their works. These models are based on different assumptions involving rock permeability (permeable, impermeable), location of the fracture initiation point (on wellbore wall, inside the rock), failure criteria (rock tensile strength, stress intensity factor, etc.), and formation condition (isotropic, anisotropic). Although different factors were considered, these models have many limitations in practical applications [17]. The actual hydraulic fracturing operation is a rather complex process, which involves a number of governing factors including reservoir and fracturing fluid properties [18].

**Table 1:** Models for predicting the fracture initiation pressure.

Model	Rock Hypothesis	Fracture Initiation	Considered Factor
Hubbert and Willis (1957) [19]	Intact and impermeable	When the circumferential effective stress on the wellbore wall reaches the rock tensile strength.	In situ stress and wellbore pressure.
Morgenstern (1962) [20]	Intact, highly permeable	When the effective stress circle first touches the failure envelope (Mohr-Coulomb) in the wall.	In situ stress and wellbore pressure.
Haimson and Fairhurst (1967) [16]	Intact, highly permeable	When the circumferential effective stress on the wellbore wall reaches the rock tensile strength.	In situ stress, wellbore pressure and poroelastic stress.
Ito and Hayashi (1991; 2008) [21,22]	Intact, permeable	When the circumferential effective stress at a point being constant distance to the wellbore wall reaches the rock tensile strength.	In situ stress, wellbore pressure, poroelastic stress, pressurization rate and wellbore diameter.
Hou et al. (2013) [23]	Intact, permeable	When a principal tensile stress exceeds the rock tensile strength.	In situ stress, wellbore pressure, wellbore orientation.
Li et al. (2016) [24]	Anisotropic	When a principal tensile stress exceeds the rock tensile strength.	In situ stress, wellbore pressure, weak plane.
Hou et al. (2017) [25]	Intact, permeable	When the maximum stress intensity factor reaches the fracture toughness of the rock at position with a certain distance from wellbore wall.	In situ stress, wellbore pressure, pore pressure and pressurization rate.
Wang et al. (2021) [26]	Intact, permeable	When the average tensile stress over the equivalent characteristic length reaches the rock tensile strength.	In situ stress, wellbore pressure.
Zhong et al. (2021) [27]	Anisotropic, permeable	When the maximum tensile stress exceeds the tensile strength of the rock matrix.	Three-dimensional stress, wellbore pressure, pore pressure, weak plane.

In recent years, many studies have focused on the effects of fluid properties on fracture initiation around wellbores. For instance, Huang et al. (2015) set preexisting fractures in storage reservoirs to simulate supercritical (SC) CO_2_ injection pressure responses for monitoring purposes by coupling thermal, hydraulic, and mechanical processes [28]. Ishida et al. (2012; 2016) and Chen et al. (2015) injected SC-CO_2_ into 17 cm granite cubes under tri-axial stresses and observed that SC-CO_2_ created more tortuous and branched fractures with lower breakdown pressures than oil and water of higher viscosity [29,30,31]. Zhang et al. (2017) conducted water, liquid CO_2_, and SC-CO_2_ fracturing on 20 cm shale cubes obtained from the outcrop of the Lower Silurian Longmaxi Formation in Sichuan Basin, China [10]. Their experiments demonstrated that the breakdown pressure of the shale samples decreased significantly with the decreasing fluid viscosity. Specifically, SC-CO_2_ fracturing was about half of that of water fracturing, which means that the parameters such as viscosity and permeability may significantly affect fracture initiation around the wellbore. Additionally, Xu et al. (2018; 2019) studied variation characteristics of gas apparent permeability for a rock matrix in high pressure reservoirs with natural fractures and established analytical models of gas apparent permeability considering shale gas flow in a fractal dual-porosity rock and flow regime in high-pressure tight sandstone reservoirs [32,33]. It can be found that apparent permeability has a certain effect on fracture initiation by affecting the fluid flow state. In addition, the stress and pore pressure variations cannot be directly measured from experiments and field studies. For this end, by using the finite element method (FEM), Li and Xing (2015) investigated the effects of parameters such as permeability on fracture initiation around the wellbore [34].

However, so far, an analytical model considering the effects of fluid properties such as viscosity has not been established due to the complexity of the hydraulic fracturing process. Moreover, in the studies mentioned above, the effect of seepage force on stress field variation has been neglected, which may lead to the result that the fracture initiation mechanism around the wellbore cannot be accurately identified. To this end, Zhou et al. (2020) proposed an analytical solution for the circumferential stress field around the wellbore formed by seepage forces and introduced this solution into the conventional computational model [35]. Zhou et al. (2021) proposed an analytical model for the circumferential stress induced by seepage force around a vertical wellbore based on linear elasticity theory and analyzed the effect of seepage force on wellbore breakdown [36]. In their study, it is usually assumed that the seepage field is under a steady state, which has a great limitation in studying the effect of seepage force because the seepage field around the wellbore varies with time during fluid injection. Therefore, studying the unsteady seepage field around the wellbore is more meaningful. In particular, for the unsteady seepage field, it is difficult to obtain the analytical solution of the stress field.

Additionally, some scholars have developed different analytical models to determine the unsteady seepage field around the wellbore. Ito and Hayashi (1991) proposed an analytical model that could predict pore pressure variation in the radial direction due to wellbore pressurization with a constant pressurization rate [21]. Yao et al. (2013) proposed a semi-analytical model based on Green’s functions and the source/sink method to facilitate the transient pressure analysis for a multi-stage fractured horizontal well in a closed box-shaped reservoir [37]. Wu et al. (2020) proposed an analytical solution to predict the variations of pore pressure and seepage force around a vertical wellbore when water is injected into the wellbore [38].

Although numerous studies were conducted to predict the seepage and stress fields around the wellbore, few studies considering the effect of seepage force under unsteady seepage have been reported. In this paper, a new seepage model that can predict the variation of pore pressure and seepage force with time around the wellbore for hydraulic fracturing was established by using the method of separation of variables and Bessel function theory, in which the flow of fluid obeys Darcy’s law. Then, a new circumferential stress calculation model considering the effect of seepage force under unsteady seepage was derived and introduced the model into the traditional stress calculation model. The proposed seepage and circumferential stress calculation models were verified by comparing them with the numerical, analytical and experimental results. The fracture initiation mechanism considering the time-dependent effect of the seepage force was analyzed and discussed based on the models proposed in this paper.

## 2. Mathematical Model for Unsteady Seepage around Vertical Wellbore

Up to now, the unsteady seepage flow model proposed by Ito and Hayashi [21] has been widely used in interpreting seepage fields during hydraulic fracturing. However, due to the complexity of the model, it is difficult to analyze the effects of some parameters, such as viscosity and permeability in the model. Therefore, a new unsteady seepage model for parametric analysis was established in this paper.

**Figure 1:**
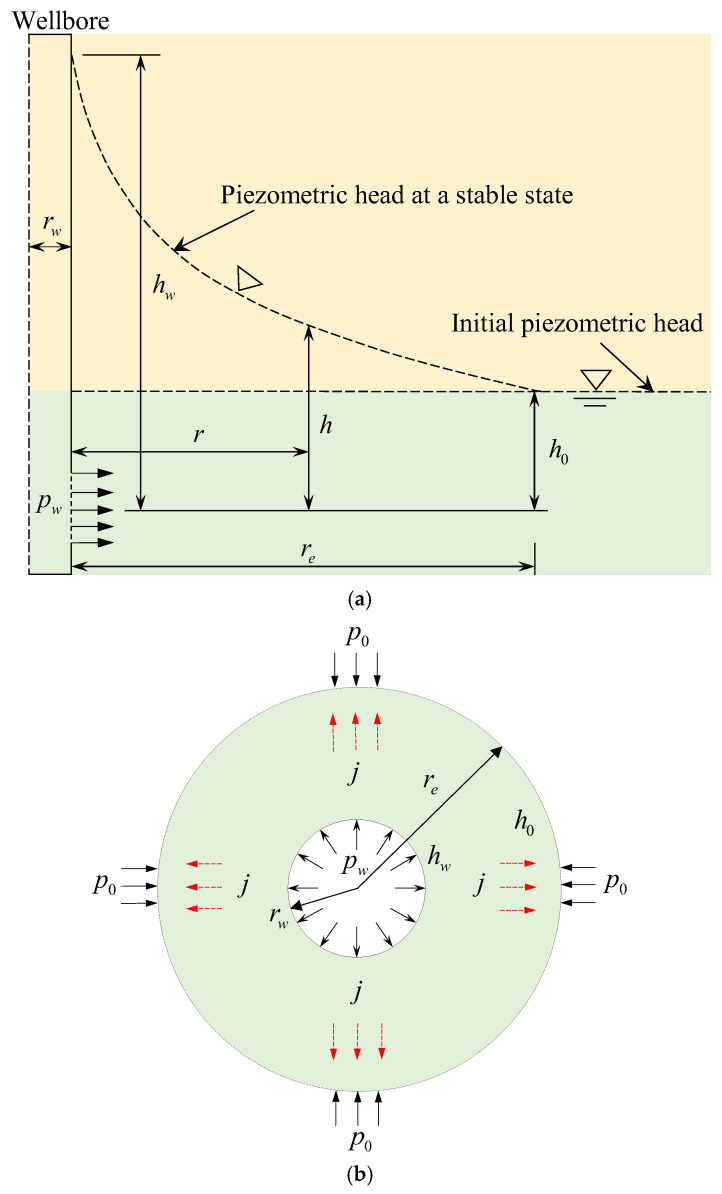
Schematic diagrams of (**a**) hydraulic head distribution around the wellbore for hydraulic fracturing and (**b**) a cross-section.

Commonly, when water is injected into a wellbore, water pressure in the wellbore will gradually increase to a pressure value, i.e., $p_{w}$ with a pressurization rate. Still, when the pressurization rate is highly great, the water pressure in the wellbore reaches a specific value instantaneously. Therefore, the water pressure in the wellbore is assumed to reach $p_{w}$ instantaneously and maintain that pressure value to simplify the problem [39]. The water pressure at a distance $r_{e}$ away from the wellbore center is fixed at a value of $p_{0}$, which is equal to the initial pore pressure. The seepage of the rock around the wellbore confirms Darcy’s law and single-phase flow, and the rock is homogeneous and isotropic. Hence, the seepage field around the wellbore for hydraulic fracturing can be treated as an axisymmetric plane problem. Figure 1 presents the diagram of seepage flow around the wellbore schematically.

For the axisymmetric plane problem, the governing equation of the unsteady seepage field is given as [39]:

(1) $$\eta \left(\frac{\partial^{2}h}{\partial r^{2}}+\frac{1}{r}\frac{\partial h}{\partial r}\right)=\frac{\partial h}{\partial t}$$

with the boundary conditions

(2) $$\{\begin{array}{c} h(r_{w},t)=h_{w}\\ h(\alpha r_{w},t)=h_{0}\\ h(r,0)=h_{0} \end{array}$$

where $h_{0}$ and $h_{w}$ denote the initial hydraulic head and the hydraulic head at the wellbore wall, respectively (m); η is the diffusion coefficient (m^2^/s), $\eta =k_{w}/s_{r}$ or η=k/(μnβ); $k_{w}$ is the hydraulic conductivity (m/s); $s_{r}$ is the specific storage (m^−1^); k is the permeability of the rock mass (m/(Pa·s)); μ is the dynamic viscosity of the fluid (Pa·s); n is the porosity of the rock (-); β is the fluid compressibility (Pa^−1^); and α is the coefficient of the radius of influence (-), $\alpha =r_{e}/r_{w}$.

Considering the relationship between pore pressure and hydraulic head, i.e., $p=\gamma_{w}\xi h$, Equation (1) can be rewritten by

(3) $$\eta \left(\frac{\partial^{2}p}{\partial r^{2}}+\frac{1}{r}\frac{\partial p}{\partial r}\right)=\frac{\partial p}{\partial t}$$

With the boundary and initial conditions

(4) $$\{\begin{array}{c} p(r_{w},t)=p_{w}\\ p(\alpha r_{w},t)=p_{0}\\ p(r,0)=p_{0} \end{array}$$

Equation (4) is no longer homogeneous, so the separation of variables cannot be applied directly. To homogenize the general boundary and initial conditions, p(r,t) can be separated in the following form:

(5) $$p(r,t)=p_{1}(r)+p_{2}(r,t)$$

where

(6) $$\{\begin{array}{c} \frac{\partial^{2}p_{1}}{\partial r^{2}}+\frac{1}{r}\frac{\partial p_{1}}{\partial r}=0\\ \begin{array}{c} p_{1}(r_{w})=p_{w}\\ p_{1}(\alpha r_{w})=p_{0} \end{array} \end{array}$$

(7) $$\{\begin{array}{c} \eta \left(\frac{\partial^{2}p_{2}}{\partial r^{2}}+\frac{1}{r}\frac{\partial p_{2}}{\partial r}\right)=\frac{\partial p_{2}}{\partial t}\\ \begin{array}{c} p_{2}(r_{w},t)=0\\ \begin{array}{c} p_{2}(\alpha r_{w},t)=0\\ p_{2}(r,0)=p_{0}-p_{1}(r) \end{array} \end{array} \end{array}$$

Equation (6) can be solved to give

(8) $$p_{1}(r)=\frac{p_{w}\ln((\alpha r_{w})/r)+p_{0}\ln(r/r_{w})}{\ln\alpha }$$

The method of separation of variables can be employed to solve Equation (7). Put

(9) $$p_{2}(r,t)=R(r)T(t)$$

Substituting Equation (9) into Equation (7) and rearranging, we obtain

(10) $$\frac{\eta }{R}\left(\frac{\partial^{2}R}{\partial r}+\frac{1}{r}\frac{\partial R}{\partial r}\right)=\frac{1}{T}\frac{\partial T}{\partial t}=-k_{n}{}^{2}$$

where $k_{n}$ denotes the eigenvalues.

Integrating Equation (10) yields

(11) $$\{\begin{array}{c} T=Ae^{-k_{n}{}^{2}t}\\ R(r)=BJ_{0}\left(\frac{k_{n}}{\sqrt{\eta }}r\right)+CY_{0}\left(\frac{k_{n}}{\sqrt{\eta }}r\right) \end{array}$$

where $J_{0}$ and $Y_{0}$ denote the Bessel functions of the first and second kind of zero order, respectively; and *A*, *B*, and *C* are the arbitrary constants.

The general solution of Equation (7) to an arbitrary $k_{n}$ is given as [40,41]:

(12) $$p_{2}(r,t)=\left[BJ_{0}\left(\frac{k_{n}}{\sqrt{\eta }}r\right)+CY_{0}\left(\frac{k_{n}}{\sqrt{\eta }}r\right)\right]Ae^{-k_{n}{}^{2}t}$$

Since the boundary conditions $p_{2}(r_{w},t)=0$, $p_{2}(\alpha r_{w},t)=0$

(13) $$\{\begin{array}{c} BJ_{0}\left(\frac{k_{n}}{\sqrt{\eta }}r_{w}\right)+CY_{0}\left(\frac{k_{n}}{\sqrt{\eta }}r_{w}\right)=0\\ BJ_{0}\left(\frac{k_{n}}{\sqrt{\eta }}\alpha r_{w}\right)+CY_{0}\left(\frac{k_{n}}{\sqrt{\eta }}\alpha r_{w}\right)=0 \end{array}$$

which is

(14) $$J_{0}\left(\frac{k_{n}}{\sqrt{\eta }}r_{w}\right)Y_{0}\left(\frac{k_{n}}{\sqrt{\eta }}\alpha r_{w}\right)-J_{0}\left(\frac{k_{n}}{\sqrt{\eta }}\alpha r_{w}\right)Y_{0}\left(\frac{k_{n}}{\sqrt{\eta }}r_{w}\right)=0$$

Put $k_{n}r_{w}/\sqrt{\eta }=x>0$, $k_{n}\alpha r_{w}/\sqrt{\eta }=\alpha x>0$, and we have

(15) $$J_{0}\left(x\right)Y_{0}\left(\alpha x\right)-J_{0}\left(\alpha x\right)Y_{0}\left(x\right)=0$$

If $x_{n}(n=1,2,3,\cdots )$ are positive roots of Equation (15), the eigenvalues can be obtained as follows:

(16) $$k_{n}{}^{2}=\eta {\left(\frac{x_{n}}{r_{w}}\right)}^{2},(n=1,2,3,\ldots )$$

Then,

(17) $$R_{n}^{0}(x_{n}r/r_{w})=J_{0}\left(x_{n}r/r_{w}\right)Y_{0}\left(x_{n}\right)-J_{0}\left(x_{n}\right)Y_{0}\left(x_{n}r/r_{w}\right)$$

The general solution to $p_{2}$ is expressed as

(18) $$p_{2}(r,t)=\sum_{n=1}^{\infty }A_{n}R_{n}^{0}(x_{n}r/r_{w})e^{-\eta {\left(\frac{x_{n}}{r_{w}}\right)}^{2}t}$$

From the initial condition

(19) $$p_{2}(r,0)=p_{0}-p_{1}(r)$$

After some manipulations, the constant is

(20) $$A_{n}=\frac{4\left(p_{0}-p_{w}\right)}{{\left(\frac{x_{n}}{r_{w}}\right)}^{2}\pi \left\{r_{w}^{2}{\left[R_{n}^{1}(r_{w})\right]}^{2}-{\left(\alpha r_{w}\right)}^{2}{\left[R_{n}^{1}(\alpha r_{w})\right]}^{2}\right\}}$$

where

(21) $$R_{n}^{1}(\alpha r_{w})=J_{1}\left(\alpha x_{n}\right)Y_{0}\left(x_{n}\right)-J_{0}\left(x_{n}\right)Y_{1}\left(\alpha x_{n}\right)$$

(22) $$R_{n}^{1}(r_{w})=J_{1}\left(x_{n}\right)Y_{0}\left(x_{n}\right)-J_{0}\left(x_{n}\right)Y_{1}\left(x_{n}\right)$$

where, $J_{1}$ and $Y_{1}$ denote the Bessel functions of the first and second kind of first order, respectively.

Combining Equations (8) and (18), the pore pressure solutions are obtained as

(23) $$p(r,t)=\frac{p_{w}\ln((\alpha r_{w})/r)+p_{0}\ln(r/r_{w})}{\ln\alpha }+\sum_{n=1}^{\infty }A_{n}R_{n}^{0}(x_{n}r/r_{w})e^{-\eta {\left(\frac{x_{n}}{r_{w}}\right)}^{2}t}$$

Equation (23) indicates the variation law of pore pressure with time in the rock mass around the wellbore. It can be found that when t → ∞, Equation (23) degenerates to the pore pressure solution under steady seepage [42].

The seepage force is expressed as a pore pressure gradient [36]. Thus, the seepage force expression can be obtained by differentiating Equation (24) concerning the independent variable r.

(24) $$j(r,t)=\frac{\left(p_{0}-p_{w}\right)}{r\ln\alpha }+\sum_{n=1}^{\infty }A_{n}\frac{dR_{n}^{0}(x_{n}r/r_{w})}{dr}e^{-k_{w}{\left(\frac{x_{n}}{r_{w}}\right)}^{2}t}$$

where

(25) $$\frac{dR_{n}^{0}(x_{n}r/r_{w})}{dr}=\frac{x_{n}}{r_{w}}\left[J_{0}\left(x_{n}\right)Y_{1}\left(x_{n}r/r_{w}\right)-J_{1}\left(x_{n}r/r_{w}\right)Y_{0}\left(x_{n}\right)\right]$$

## 3. Circumferential Stress Calculation Model Considering the Effect of Seepage Force

**Figure 2:**
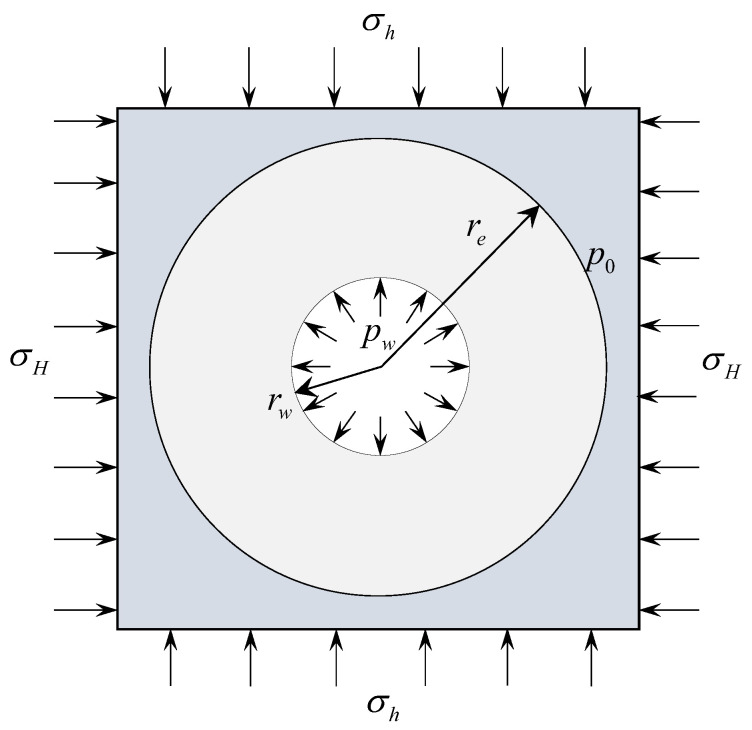
Mechanical model of the vertical wellbore.

As shown in Figure 2, two horizontal principal stresses $\sigma_{H}$ and $\sigma_{h}$ are applied to the external boundaries of the vertical wellbore with radius $r_{w}$, internal water pressure $p_{w}$ and initial pore pressure $p_{0}$. The seepage field in the reservoir is assumed as in Section 2. Compression stress and pore pressure are assumed to be positive in this paper.

### 3.1. Traditional Model (Point Stress Model)

When the reservoir rock is impermeable and isotropic, the stress state around the wellbore can be derived according to the principle of superposition as follows [43]:

(26) $$\sigma_{r}=\frac{\sigma_{H}+\sigma_{h}}{2}\left(1-\frac{r_{w}{}^{2}}{r^{2}}\right)+\frac{\sigma_{H}-\sigma_{h}}{2}\left(1-\frac{4r_{w}{}^{2}}{r^{2}}+\frac{3r_{w}{}^{4}}{r^{4}}\right)\cos2\theta +p_{w}\frac{r_{w}{}^{2}}{r^{2}}$$

(27) $$\sigma_{\theta }=\frac{\sigma_{H}+\sigma_{h}}{2}\left(1+\frac{r_{w}{}^{2}}{r^{2}}\right)-\frac{\sigma_{H}-\sigma_{h}}{2}\left(1+\frac{3r_{w}{}^{4}}{r^{4}}\right)\cos2\theta -p_{w}\frac{r_{w}{}^{2}}{r^{2}}$$

(28) $$\tau_{r\theta }=\frac{\sigma_{h}-\sigma_{H}}{2}\left(1+\frac{2r_{w}{}^{2}}{r^{2}}-\frac{3r_{w}{}^{4}}{r^{4}}\right)\sin2\theta$$

where $\sigma_{r}$, $\sigma_{\theta }$ and $\tau_{r\theta }$ are the radial stress, circumferential stress, and shear stress of a point (r, θ) in the cylindrical coordinate system (MPa); *r* is the distance from the point to the wellbore center (r > $r_{w}$) (m); θ is the angle measured counter-clockwise from the radius in the direction of $\sigma_{H}$ ($\sigma_{H}$ direction corresponds to θ = 0) (-); $\sigma_{H}$ is the maximum horizontal effective principal stress (MPa), and $\sigma_{h}$ is the minimum horizontal effective principal stress (MPa).

Research results show that fractures are mainly formed in the direction of $\sigma_{H}$, i.e., θ = 0 or π during hydraulic fracturing [18]. Therefore, the circumferential stresses at θ = 0 or π are mainly discussed in this study.

When the reservoir rock is permeable, the circumferential stress around the wellbore during hydraulic fracturing can be decomposed into three elastic stress states [18]. The three stress components are: (1) a in situ circumferential stress component ($S^{1}{}_{\theta }$) due to the two horizontal principal stresses, (2) a circumferential stress component developed due to the injection pressure of the fracturing fluid in the wellbore ($S^{2}{}_{\theta }$) and (3) a circumferential stress component generated due to the pore pressure distribution through the rock mass ($S^{3}{}_{\theta }$).

(29) $$\sigma_{\theta }=S^{1}{}_{\theta }+S^{2}{}_{\theta }+S^{3}{}_{\theta }$$

where

(30) $$S^{1}{}_{\theta }=\frac{\sigma_{H}+\sigma_{h}}{2}\left(1+\frac{r_{w}{}^{2}}{r^{2}}\right)-\frac{\sigma_{H}-\sigma_{h}}{2}\left(1+\frac{3r_{w}{}^{4}}{r^{4}}\right)\cos2\theta$$

(31) $$S^{2}{}_{\theta }=-p_{w}\frac{r_{w}{}^{2}}{r^{2}}$$

(32) $$S^{3}{}_{\theta }=\frac{\alpha_{B}(1-2\nu )}{\left(1-\nu \right)}\left[\left[p(r,t)-p_{0}\right]-\frac{1}{r^{2}}\int_{r_{w}}^{r}\left[p(r,t)-p_{0}\right]rdr\right]$$

in which

(33) $$\alpha_{B}=1-\frac{C_{m}}{C_{b}}=1-\frac{K_{b}}{K_{m}}$$

(34) $$p\left(r,t\right)=C\int_{0}^{t}f\left(r,s\right)ds+p_{0}$$

(35) $$f\left(r,t\right)=1+\int_{0}^{\infty }\exp(-\eta u^{2}t)\left[\frac{J_{0}(ur)Y_{0}(ur_{w})-Y_{0}(ur)J_{0}(ur_{w})}{J_{0}{\left(ur_{w}\right)}^{2}+Y_{0}{\left(ur_{w}\right)}^{2}}\right]\frac{du}{u}$$

where ν is the Poisson’s ratio of the reservoir rock (-); and $\alpha_{B}$ is Biot’s poro-elastic coefficient related to the pore structure of reservoir rocks (-). *C_b_* and *K_b_* are the compression coefficient (MPa^−1^) and bulk modulus of the rock framework (MPa), respectively, while *C_m_* and *K_m_* are the compression coefficient (MPa^−1^) and bulk modulus of the mineral matrix (MPa), respectively. The function p(r,t) represents the pore pressure distribution due to hydraulic fracturing with constant pressurization rate *C*. If the constant pressurization rate is large, p(r,t) is equal to $p_{0}$ and $S^{3}{}_{\theta }$ is expressed as

(36) $$S^{3}{}_{\theta }=0$$

At this time, the circumferential stress is given as follows:

(37) $$\sigma_{\theta }=S^{1}{}_{\theta }+S^{2}{}_{\theta }$$

If the pressurization rate approaches zero, p(r,t) is equal to $p_{w}$ and $S^{3}{}_{\theta }$ is represented by

(38) $$S^{3}{}_{\theta }=\frac{\alpha_{B}}{2}\frac{1-2\nu }{1-\nu }\left(1+\frac{r_{w}{}^{2}}{r^{2}}\right)(p_{w}-p_{0})$$

The circumferential stress at the wellbore wall is given as follows:

(39) $$\sigma_{\theta }=(\sigma_{H}+\sigma_{h})-2(\sigma_{H}-\sigma_{h})\cos2\theta -p_{w}+\alpha_{B}\frac{1-2\nu }{1-\nu }(p_{w}-p_{0})$$

#### 3.1.1. Hubbert-Willis Criterion (H-W Criterion)

The H-W criterion proposed by Hubberts and Willis [19] is the first theoretical criterion to predict breakdown pressure during hydraulic fracturing, in which hydraulic fractures are assumed to be created around the wellbore when the effective circumferential stress reaches the rock tensile strength $\sigma_{t}$. The H-W criterion is given by

(40) $$\sigma_{\theta }=S^{1}{}_{\theta }+S^{2}{}_{\theta }-p_{0}=-\sigma_{t}$$

Substituting Equations (30) and (31) into Equation (40), the breakdown pressure $p_{b}$ can be obtained as follows:

(41) $$p_{b}=3\sigma_{h}-\sigma_{H}-p_{0}+\sigma_{t}$$

#### 3.1.2. Haimson–Fairhurst Criterion (H-F Criterion)

The H-F criterion [16] is derived based on Biot’s poro-elastic theory. It is assumed that the fluid penetrates into the rock mass, and hydraulic fracture occurs at the wellbore wall. The H-F criterion is established as follows:

(42) $$\sigma_{\theta }=S^{1}{}_{\theta }+S^{2}{}_{\theta }+S^{3}{}_{\theta }-p_{w}=-\sigma_{t}$$

The breakdown pressure $p_{b}$ can be represented by

(43) $$p_{b}=\frac{3\sigma_{h}-\sigma_{H}+\sigma_{t}-\alpha_{B}\left(\frac{1-2\nu }{1-\nu }\right)p_{0}}{\left(2-\alpha_{B}\frac{1-2\nu }{1-\nu }\right)}$$

If the initial pore pressure is zero, Equation (43) is rewritten by

(44) $$p_{b}=\frac{3\sigma_{h}-\sigma_{H}+\sigma_{t}}{\left(2-\alpha_{B}\frac{1-2\nu }{1-\nu }\right)}$$

### 3.2. Deviation of Circumferential Stress Calculation Model Considering the Time-Dependent Effect of Seepage Force during Hydraulic Fracturing

In previous studies, $S^{2}{}_{\theta }$ is calculated using wellbore pressure as a surface force when fracturing fluid penetrates into reservoir rocks. However, $S^{2}{}_{\theta }$ should be replaced with the circumferential stress $S^{4}{}_{\theta }$ formed by the seepage force when the effect of the seepage force is considered. In this case, the wellbore pressure at the wellbore wall is treated as pore pressure. The expression of $S^{4}{}_{\theta }$ can be derived by using the seepage solution proposed in Section 2.

The equilibrium equation is given as follows:

(45) $$\frac{\partial \sigma_{r}}{\partial r}+\frac{\sigma_{r}-\sigma_{\theta }}{r}+j(r,t)=0$$

with the boundary conditions

(46) $$\{\begin{array}{c} \sigma_{r}=0,r=r_{w}\\ \sigma_{r}=0,r=r_{e} \end{array}$$

Combining the above equations, circumferential stress formed by the seepage force under unsteady seepage can be obtained as

(47) $$S^{4}{}_{\theta }=\frac{1}{r^{2}}\frac{1-2\nu }{1-\nu }\{\frac{r_{w}^{2}+r^{2}}{r_{w}^{2}-r_{e}^{2}}\left[\int_{r_{a}}^{r_{e}}p(r,t)rdr+\frac{1-\nu }{1-2\nu }r_{e}^{2}\left(p_{a}-p_{0}\right)\right]-\;\int_{r_{a}}^{r}p(r,t)rdr\}-\frac{\nu }{1-\nu }p\left(r,t\right)+p_{a}$$

If the seepage field reaches a stable state, *S*^4^*_θ_* is reduced to the solution proposed by Zhou et al. (2021) as follows [36]:

(48) $$S^{4}{}_{\theta }=\frac{p_{a}-p_{0}}{2(1-\nu )}\left[\frac{\ln r-\ln r_{w}+2\nu -1}{\ln r_{e}-\ln r_{w}}-\frac{r_{e}^{2}(r^{2}+r_{w}^{2})}{r^{2}(r_{e}^{2}-r_{w}^{2})}\right]$$

When the rock is impermeable, the circumferential stress field around the wellbore can be determined as follows:

(49) $$\sigma_{\theta }=S^{1}{}_{\theta }+S^{2}{}_{\theta }$$

When the rock is permeable, $\sigma_{\theta }$ can be expressed as

(50) $$\sigma_{\theta }=S^{1}{}_{\theta }+S^{3}{}_{\theta }+S^{4}{}_{\theta }-p(r,t)$$

If fractures are assumed to initiate on the wellbore wall, the lower limit of initiation pressure can be calculated based on Equation (50).

(51) $$p_{b}=\frac{3\sigma_{h}-\sigma_{H}+\sigma_{t}}{1-\frac{1}{2(1-\nu )}\left(\frac{2\nu -1}{\ln\alpha }-\frac{2r_{e}{}^{2}}{r_{e}{}^{2}-r_{w}{}^{2}}\right)}$$

If the effect of seepage force is ignored, the circumferential stress around the wellbore can be expressed according to the model proposed by Ito (2008) [22] as follows:

(52) $$\sigma_{\theta }=S^{1}{}_{\theta }+S^{2}{}_{\theta }+S^{3}{}_{\theta }-p(r,t)$$

## 4. Model Verification

### 4.1. Verification of Unsteady Seepage Model

**Figure 3:**
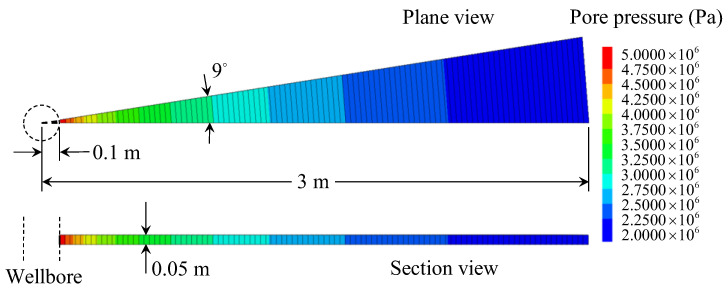
Pore pressure contours of the numerical calculation (FLAC3D).

**Figure 4:**
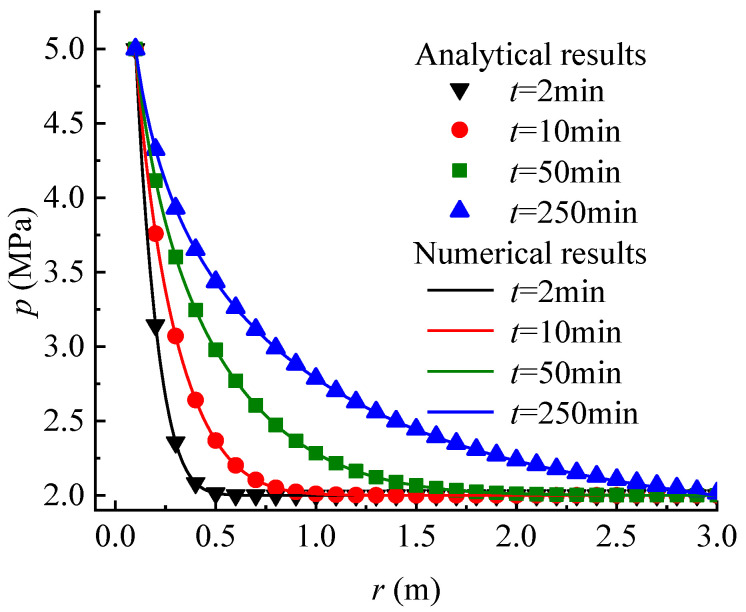
Comparison of the pore pressure versus distance from the wellbore wall between the analytical and the numerical results (FLAC3D) for various times.

**Figure 5:**
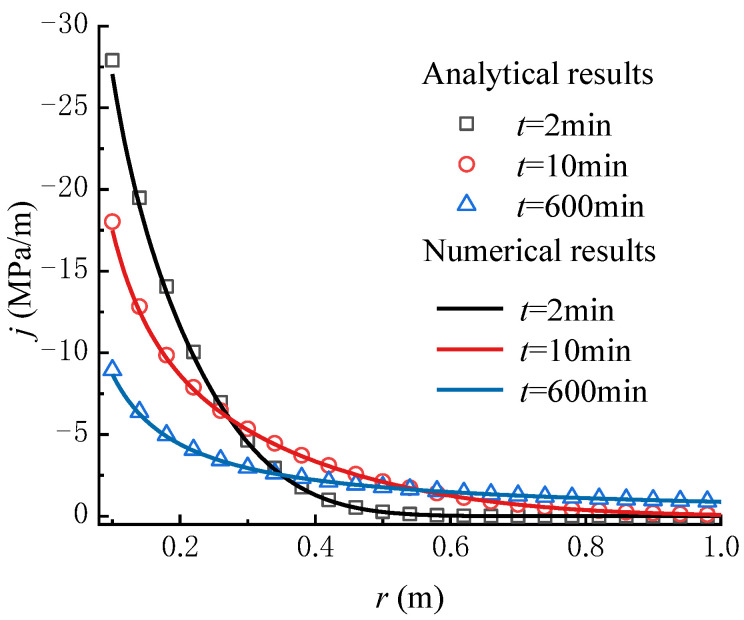
Comparison of the seepage force versus distance from the wellbore wall between the analytical and numerical results (FLAC3D) for various times.

To verify the correctness of the seepage model proposed in this paper, a comparison with the numerical results calculated by the FLAC3D program code is made. The MATLAB program is compiled to carry out the calculation of the proposed analytical model. The main parameters employed in the calculation are set as: $k_{w}$ = 1 × 10^−7^ m/s, $s_{r}$ = 1 × 10^−3^ m^−1^, $r_{w}$ = 0.1 m, $p_{0}$ = 2 MPa, $p_{w}$ = 5 MPa, and α = 30. The other parameters used in the numerical calculation are given in Table 2. The pore pressure at the wellbore wall is fixed to the value of $p_{w}$, and the value of $p_{0}$ at a distance $r_{e}$ from the wellbore center. Except for the inner and outer faces, all the boundaries are set to zero flux and are impermeable by default. The total zones of 100 are used, lined up, and graded in the radial direction. As a result, the calculation condition employed in the numerical model is the same as that in the analytical model. Figure 3 shows the pore pressure contours obtained by numerical calculation. Figure 4 and Figure 5 compare the pore pressure and seepage force variations between the analytical and numerical results, respectively. As shown in Figure 4, the greater the distance from the wellbore wall, the smaller the pore pressure in the rock mass. In addition, as time goes on, the pore pressure at each point in the rock mass gradually increases until reaching a steady state. Meanwhile, as shown in Figure 5, the seepage force within a certain range near the wellbore wall gradually decreases with time and distance from the wellbore wall until reaching a steady state. Conversely, the seepage force away from the wellbore wall is smaller than that near the wellbore wall and increases slightly with time. The analytical results match well with the numerical results. It means that the proposed model is reliable in predicting the variations of pore pressure and seepage force in rock mass around the wellbore.

**Figure 6:**
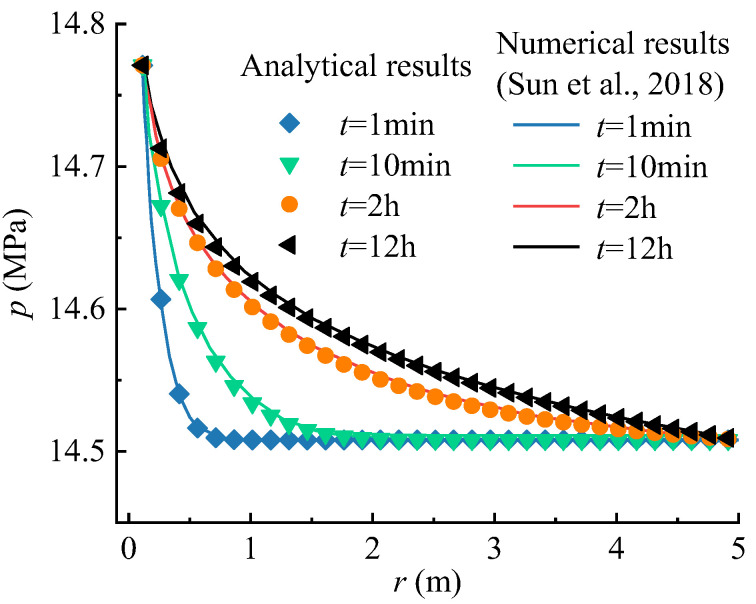
Comparison between the results from the model in this paper and the numerical one (Sun et al., 2018) [44] of the pore pressure versus distance from the wellbore wall for different times.

The applicability of the proposed seepage model in practice engineering is validated by comparing it with the numerical results in previous work [44]. Sun et al. (2018) simulated the variation of pore pressure with time around an exploration well performed in the Shenhu area of the South China Sea by using the multiphysics simulator TOUGH + HYDRATE [44]. This software is widely used to simulate gas recovery from hydrate reservoirs in marine or permafrost regions [45]. The parameters provided by the example of the Shenhu area are shown in Table 3. The geometric and boundary conditions in the analytical model are the same with the numerical model performed by Sun et al. (2018) [44]. Figure 6 shows the comparison in pore pressure between the two models. The results show that the pore pressure distributions from the two models are very consistent.

### 4.2. Verification of Circumferential Stress Calculation Model

Zhou et al. (2021) studied the effect of seepage force on the circumferential stress field under steady seepage [36]. The difference between their study and that of this paper is that Zhou et al. (2021) only analyzed the effect of seepage force under steady seepage. However, in our study, in addition to the effect of seepage force under steady seepage, the effect of seepage force under unsteady seepage was also considered. Therefore, it can be found that the circumferential stress calculation model by Zhou et al. (2021) is a special case of our model. The parameters used in the comparison are listed in Table 4.

**Figure 7:**
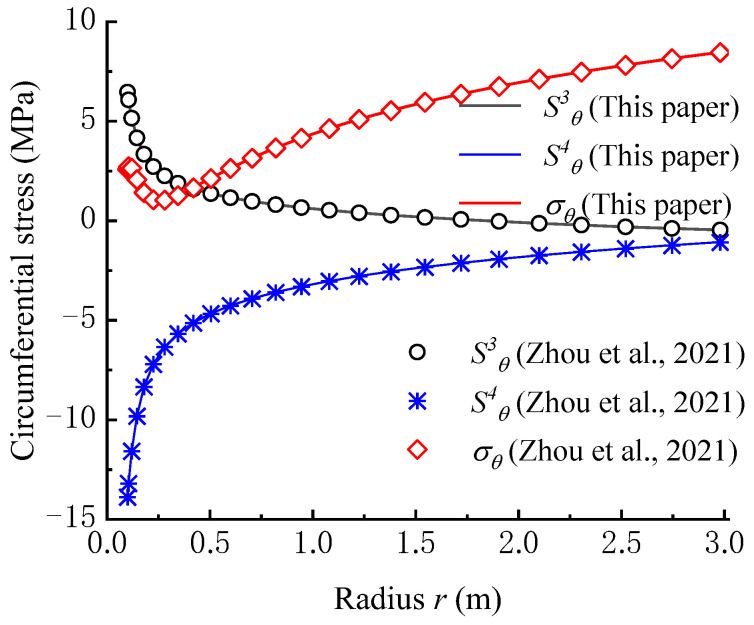
Verification of circumferential stress calculation model [36].

According to the seepage condition, when the elapsed time exceeds ten days, the seepage field reaches a steady state. Hence, in the calculation of our model, the time is set as 10 days. As shown in Figure 7, the calculation results of $\sigma_{\theta }$, $S^{3}{}_{\theta }$, and $S^{4}{}_{\theta }$ from our model are perfectly coincident with that from the model proposed by Zhou et al. (2021). The calculation error between the two models is below 1%.

To verify the applicability of the circumferential stress calculation model proposed in this paper, the experimental results reported by Wang et al. (2017) [46] are employed. In their experiment, SC-CO_2_ fluid was used as a fracturing fluid and the injection rate was lower than 80 mL/min, and then Niobrara shale cubes of 20 cm from Colorado were employed. As the viscosity of SC-CO_2_ fluid is highly low, the seepage process of fluid during a fluid injection can be regarded as a steady state. In this case, the breakdown pressures of samples can be obtained from Equation (52). Table 5 shows a comparison between the experimental and predicted results. It can be found that the results calculated by using the H-F criterion (Equation (44)) are generally larger than those measured in the experiment. This means that the conventional model may overestimate the breakdown pressure of rocks during injection of fluid such as SC-CO_2_ fluid. However, the breakdown pressures calculated from Equation (52) are relatively consistent with the experimental results, except for sample 2, where the calculation value is much lower than that measured in the experiment. If the difference between the initiation and breakdown pressures is considered [42], the predicted result in sample 2 is reliable. As recently reported, when SC-CO_2_ fluid is used as a fracturing fluid, the observed initiation pressure is lower than other fluids [11,46]. This shows that the effect of seepage force must be considered in interpreting the seepage field. Therefore, it can be found that the circumferential stress calculation model proposed in this paper can be used to study the mechanism of fracture initiation.

## 5. Results and Discussion

In this section, the time-dependent effect of seepage force on the mechanism of fracture initiation is analyzed intensively with the consideration of different parameters such as time, hydraulic conductivity and fluid viscosity. The calculation condition listed in Table 4 for analysis is employed.

### 5.1. Variation of Circumferential Stress Field with Time

The variations of pore pressure and seepage force with time were analyzed in Section 4.1. Due to the variations of pore pressure and seepage force, the circumferential stresses $S^{3}{}_{\theta }$ and $S^{4}{}_{\theta }$ created by the poro-elastic stress and seepage force, respectively, will vary with time. Thus, the variations of $S^{3}{}_{\theta }$, $S^{4}{}_{\theta }$ and $\sigma_{\theta }$ are analyzed in relationship with time.

**Figure 8:**
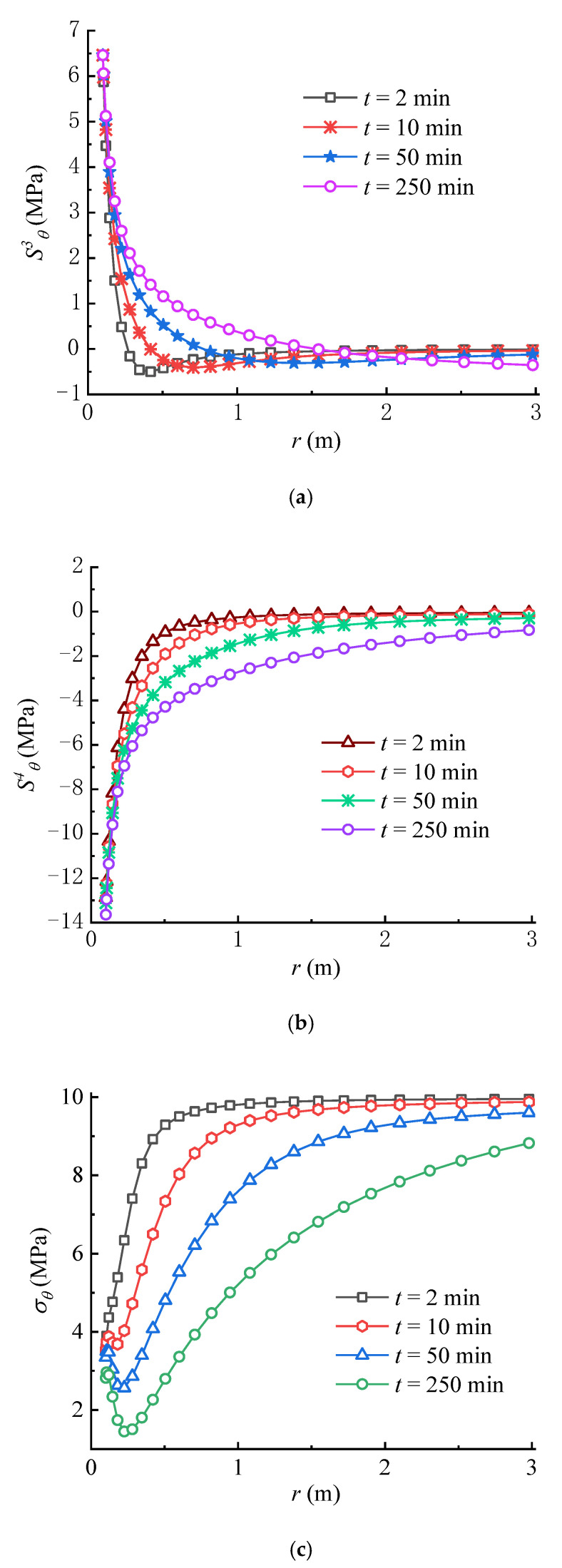
Variations of (**a**) $S^{3}{}_{\theta }$, (**b**) $S^{4}{}_{\theta }$ and (**c**) $\sigma_{\theta }$ with time ($p_{w}$ = 15 MPa).

**Figure 9:**
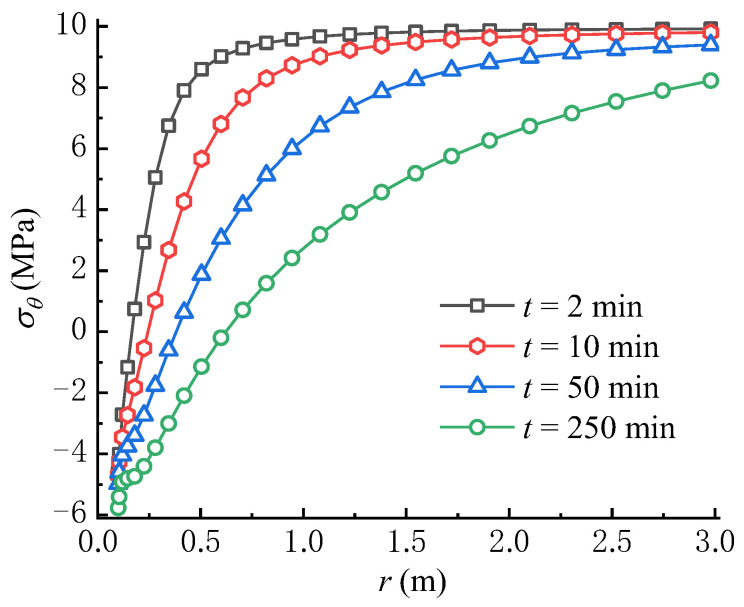
Variation of $\sigma_{\theta }$ with time ($p_{w}$ = 20 MPa).

From Figure 8a,b, it can be seen that $S^{3}{}_{\theta }$ and $S^{4}{}_{\theta }$ overall increase over time, in which the circumferential stress formed by the seepage force is negative. The variation rule of $S^{4}{}_{\theta }$ is contrary to that of the seepage force, which decreases with time near the wellbore wall. From the perspective of fracture initiation, it is obvious that $S^{4}{}_{\theta }$ facilitates the tensile failure of rock, while $S^{3}{}_{\theta }$ inhibits the rock failure, which is consistent with previous studies [22,36]. As shown in Figure 8c, the total circumferential effective stress $\sigma_{\theta }$, which is calculated from Equation (50), decreases over time because the increase rate of the circumferential stress induced by seepage force is larger than that created by the poroelastic stress. Moreover, the increase of pore pressure in the rock mass with time lowers $\sigma_{\theta }$ according to Terzaghi’s effective stress principle. Significantly, the concave part in the total circumferential effective stress curve tends to decline over time; namely, the total circumferential effective stress not far away from the wellbore wall is lower than that on the wellbore wall. If the tensile strength of rock is equal to zero or very small, the tensile failure of rock may first occur within the rock mass rather than on the wellbore wall, which is consistent with the results in previous studies [17,22]. Meanwhile, if the tensile strength of rock is larger, the rock failure may first occur on the wellbore wall rather than within the rock mass. In this case, the tensile failure of rock requires a higher wellbore pressure, i.e., $p_{w}$ = 20 MPa, as shown in Figure 9. Consequently, there exists a critical wellbore pressure, which indicates that when the wellbore pressure is smaller than the critical pressure, the tensile failure of rock may occur within the rock mass. It can be found that depending on the condition, the fracture may be initiated on the wellbore wall or within the rock mass.

**Figure 10:**
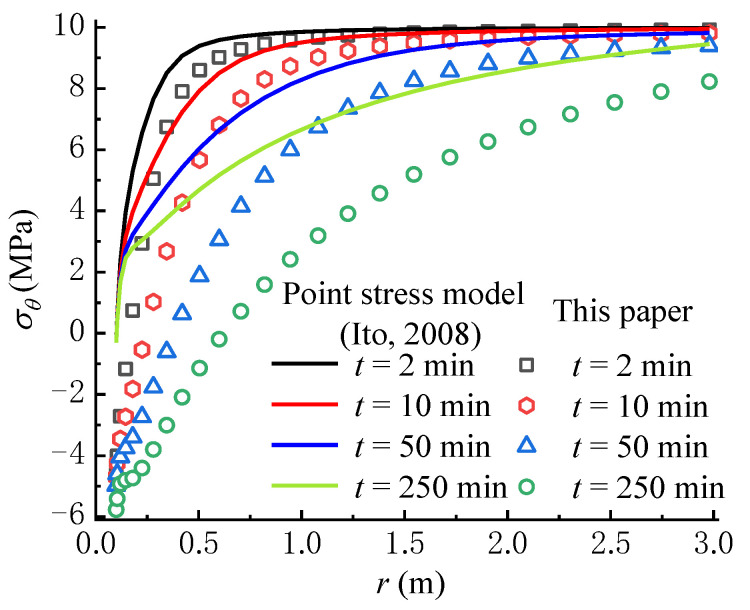
Comparison with the point stress model (Ito, 2008) [22].

Figure 10 compares the variations of total circumferential effective stresses predicted by the point stress model [22] and the model proposed in this paper. The results from the point stress model are larger than that from our model, especially the differences between the two models, which are significantly large in the vicinity of the wellbore wall. This means that the seepage force significantly impacts the total circumferential effective stress field during fluid injection, because the model proposed in this paper is obtained by replacing $S^{2}{}_{\theta }$ in the point stress model with $S^{4}{}_{\theta }$.

### 5.2. Variation of Circumferential Stress Field with the Hydraulic Conductivity

The hydraulic conductivity is an important factor not only in studying the characteristics of fluid flow, but also in the analysis of the stress field during hydraulic fracturing [47]. To investigate the variations of circumferential stress fields under different permeability conditions, the hydraulic conductivity $k_{w}$ employed in analysis is set as 10^−9^ m/s, 10^−8^ m/s, 10^−7^ m/s and 10^−6^ m/s, respectively.

**Figure 11:**
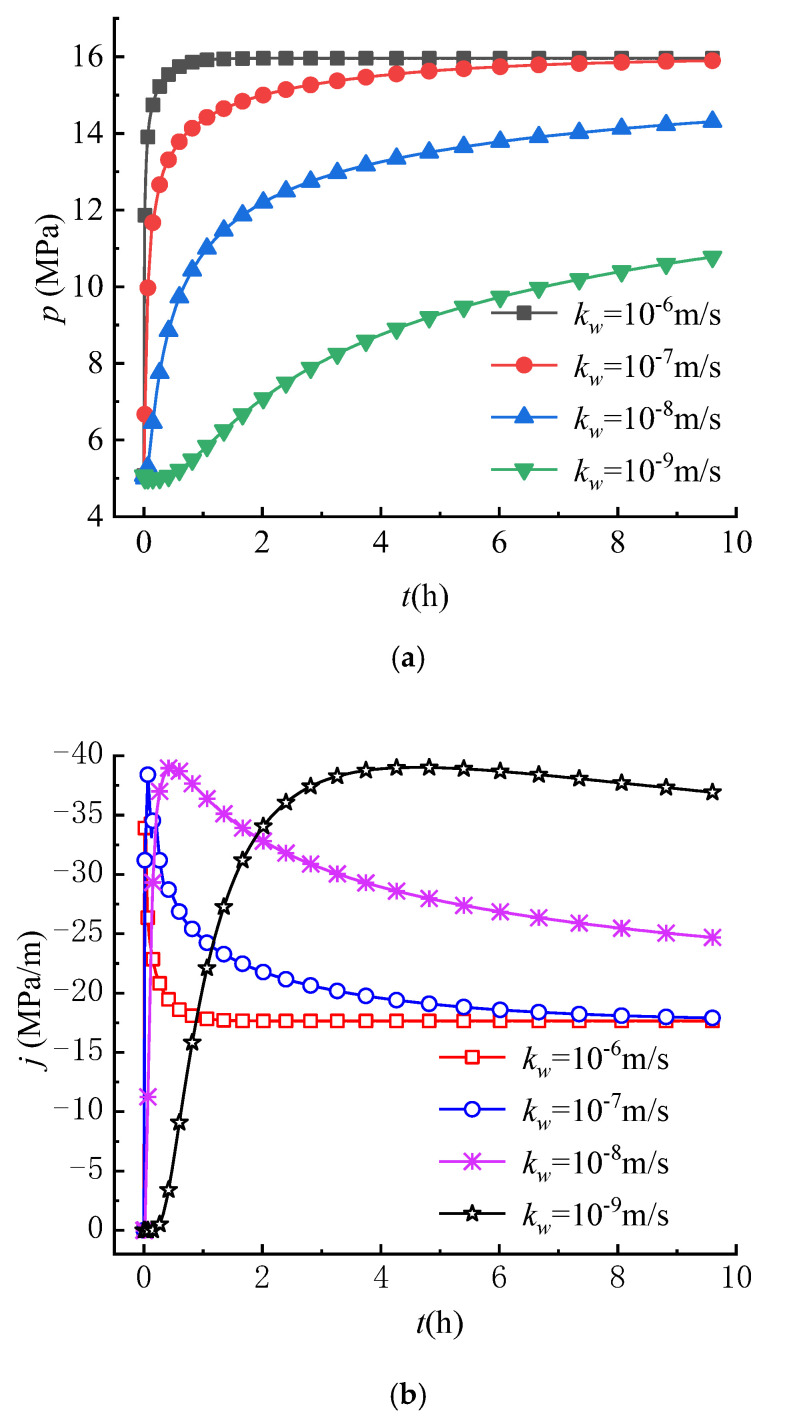

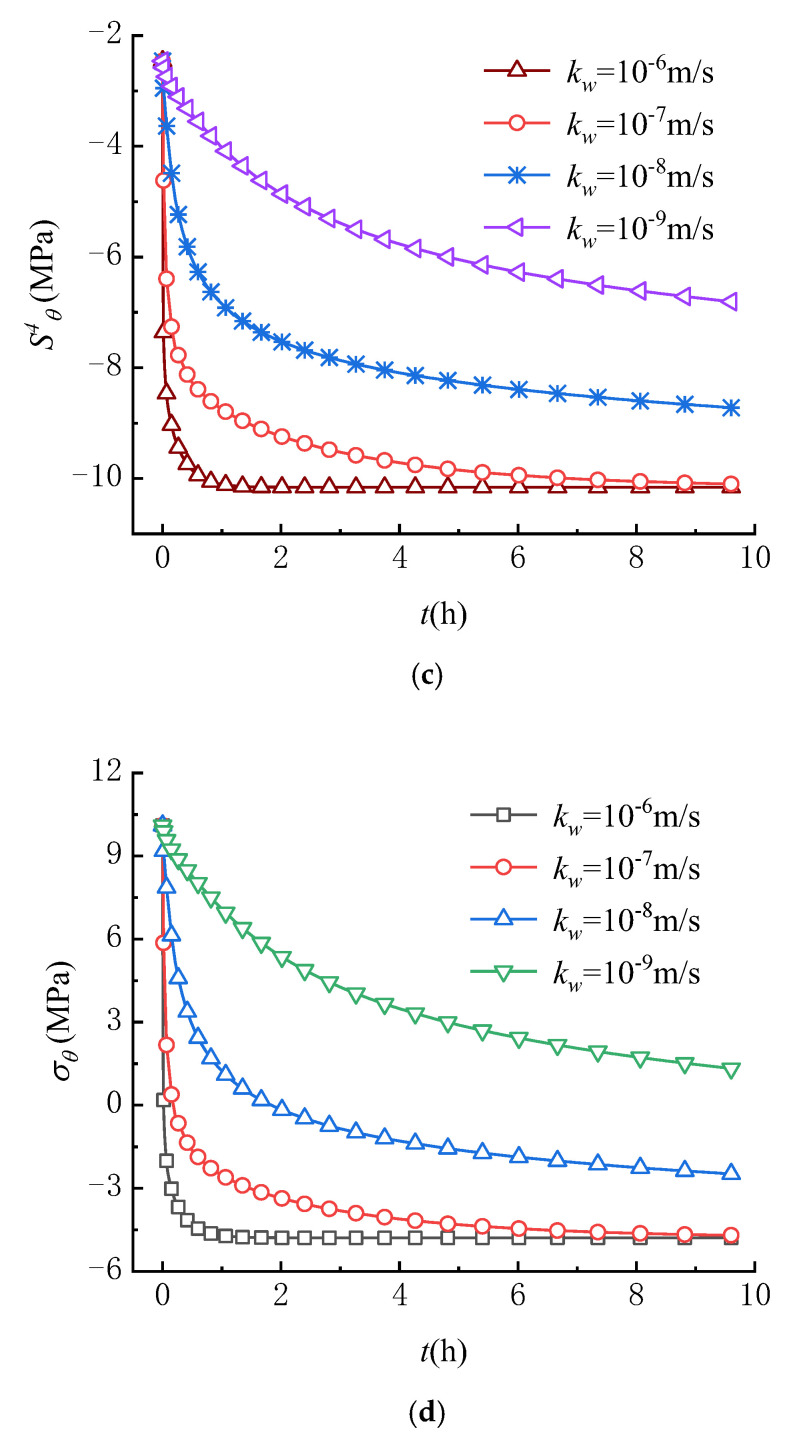
Variations of (**a**) p, (**b**) j, (**c**) $S^{4}{}_{\theta }$ and (**d**) $\sigma_{\theta }$ with different hydraulic conductivity (*r* = 0.25 m).

Figure 11 shows the effects of the hydraulic conductivity on the variations of pore pressure, seepage force, circumferential stress induced by seepage force and total circumferential effective stress with time at r = 0.25 m. As shown in Figure 11a, the greater the hydraulic conductivity, the faster the pore pressure increases over time, and the shorter the time the pore pressure reaches a steady state. It can be found that the hydraulic conductivity only affects the increase rate of the pore pressure. The effect of the hydraulic conductivity on the variation of seepage force is shown in Figure 11b. The results show that the greater the hydraulic conductivity, the faster the seepage force increases to the maximum value, then gradually decreases with time and reaches a stable state in a shorter time. These variation laws of pore pressure and seepage force with time are consistent with the study results of Wu et al. (2020) [38].

As shown in Figure 11c,d, the variations of $S^{4}{}_{\theta }$ and $\sigma_{\theta }$ with time are similar, indicating that the variation of $\sigma_{\theta }$ mainly depends on $S^{4}{}_{\theta }$. When the hydraulic conductivity is larger, $S^{4}{}_{\theta }$ and $\sigma_{\theta }$ decrease more quickly over time until the seepage field reaches a steady state. Therefore, tensile failure of rocks is more likely to occur in rocks with large hydraulic conductivity than in rocks with small hydraulic conductivity.

### 5.3. Variation of Circumferential Stress Field with Fluid Viscosity

Some experimental results show that fluid viscosity has an important impact on the initiation or breakdown pressures of rock [11,46]. Although fluid viscosity was earlier introduced into the point stress model, the effect of fluid viscosity on fracture occurrence was not adequately analyzed due to the limitations of the model, which did not take into account the effect of seepage forces. For this reason, the circumferential stress fields under different viscosity conditions are compared in this paper. The fluid viscosity employed in analysis is set as: μ = 10^−2^, 10^−3^, 10^−4^, 10^−5^ Pa∙s.

**Figure 12:**
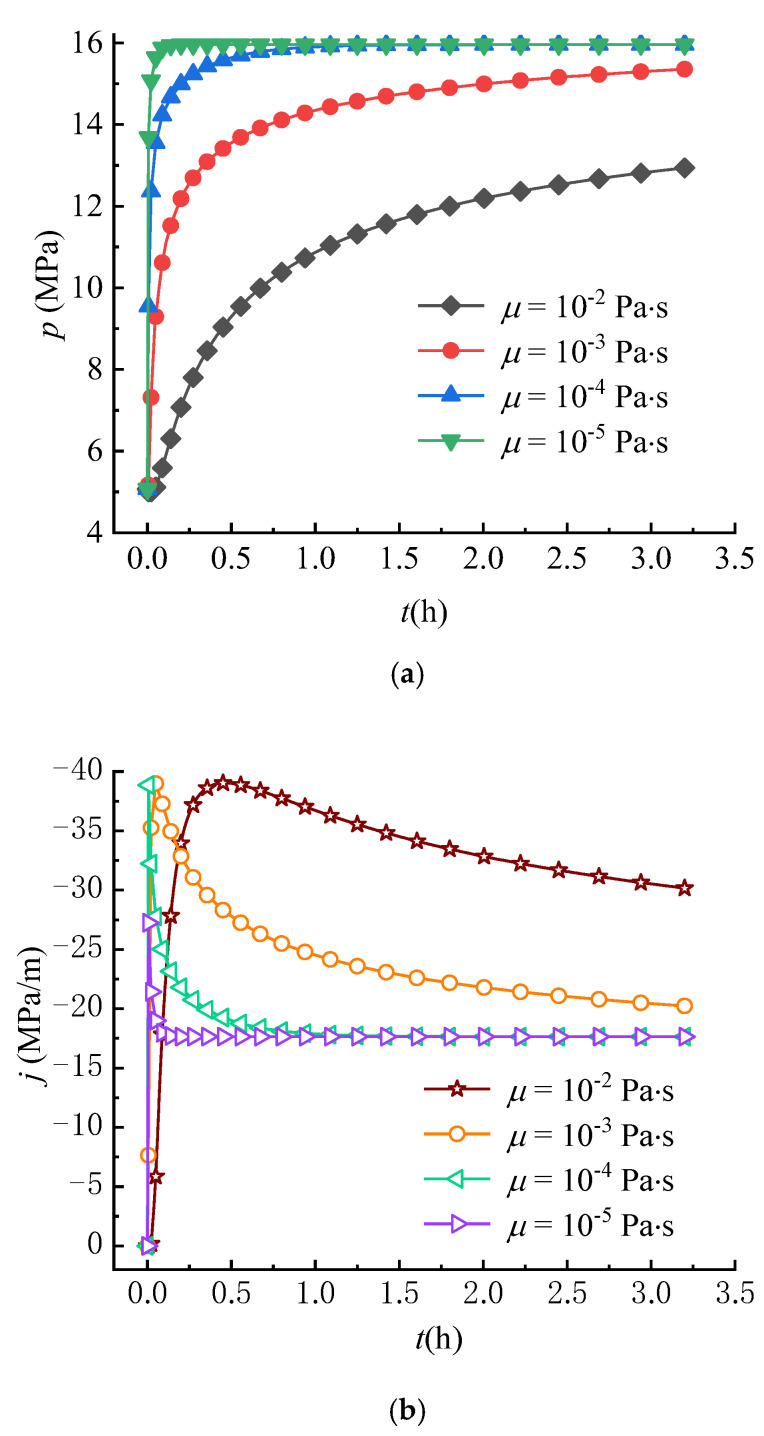

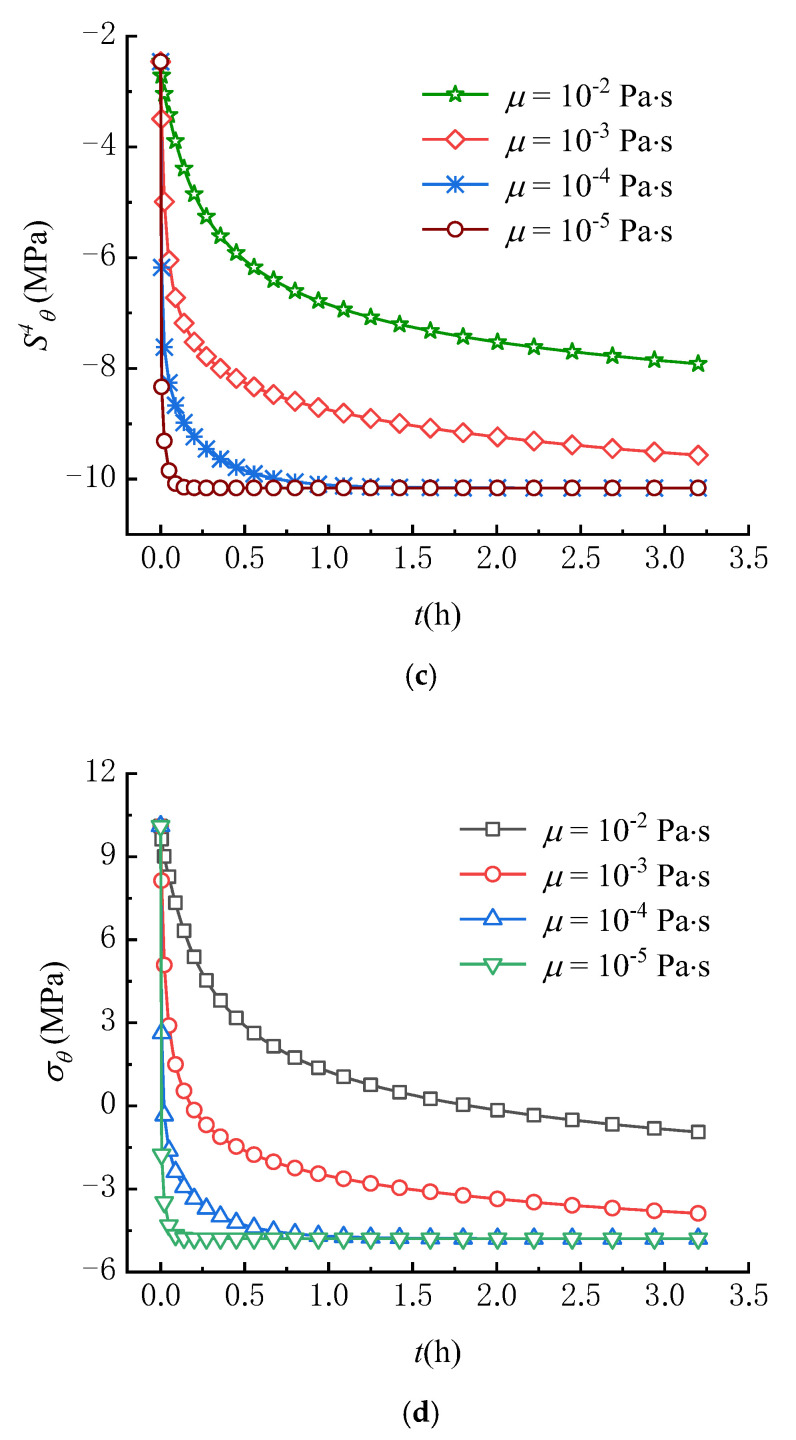
Variations of (**a**) p, (**b**) j, (**c**) $S^{4}{}_{\theta }$ and (**d**) $\sigma_{\theta }$ with different viscosities (*r* = 0.25 m).

Figure 12a,b show the effect of viscosity on the variations of pore pressure and seepage force at *r* = 0.25 m. The pore pressure increases over time until reaching a steady state, where the larger the fluid viscosity, the slower the increase rate of pore pressure. Meanwhile, the larger the fluid viscosity, the more slowly the seepage force reaches a steady state. As a result, the effect of viscosity on the pore pressure and seepage force is opposite to that of the hydraulic conductivity. Thus, the effect of viscosity on $S^{4}{}_{\theta }$ and $\sigma_{\theta }$ is also opposite to that of the hydraulic conductivity as shown in Figure 12c,d, i.e., the larger the fluid viscosity, the more slowly $S^{4}{}_{\theta }$ and $\sigma_{\theta }$ decrease. It can be found that the lower the fluid viscosity, the faster the tensile failure of rock occurs because $\sigma_{\theta }$ decreases more quickly over time. These results can well-explain the reason that the lower breakdown pressure is observed in the experiments using fluids with lower viscosity (such as liquid CO_2_) as fracturing fluids [30,31].

## 6. Conclusions

In this study, a new seepage model for predicting the variations of pore pressure and seepage force with time around a vertical wellbore and a new circumferential stress calculation model considering the time-dependent effect of seepage force were established. Then, the fracture initiation mechanism around the wellbore was investigated. The conclusions are summarized as follows:

- The comparison with the experimental results obtained by using the SC-CO_2_ fluid indicates that the circumferential stress calculation model considering the time-dependent effect of seepage force can predict more accurately the fracture initiation pressure compared to the existing model.
- During hydraulic fracturing, the circumferential stress $S^{3}{}_{\theta }$ formed by poroelastic stress inhibits the fracture initiation and the circumferential stress $S^{4}{}_{\theta }$ induced by seepage force facilitates the fracture initiation because $S^{3}{}_{\theta }$ is mainly compression stress and $S^{4}{}_{\theta }$ is tensile stress. In addition, the increase rate of $S^{4}{}_{\theta }$ over time is larger than that of $S^{3}{}_{\theta }$.
- When wellbore pressure is constant, the total circumferential effective stress is $\sigma_{\theta }$, considering the effect of seepage force decreases over time, namely becoming tensile stress. The longer the time the fluid penetrates the rock mass, the greater the possibility of fracture initiation. Particularly, when the tensile strength of the rock is smaller, the tensile failure of the rock will be initiated within the rock mass rather than on the wellbore wall.
- For different hydraulic conductivities, when the hydraulic conductivity is lower, more time is needed for the distributions of pore pressure, seepage force, and circumferential stresses $S^{3}{}_{\theta }$, $S^{4}{}_{\theta }$ and $\sigma_{\theta }$ to reach a steady state. It means that the lower the hydraulic conductivity, the later the fracture initiation occurs.
- For different fluid viscosities, when the fluid viscosity is lower, a shorter time is needed for the distributions of pore pressure, seepage force and circumferential stresses $S^{3}{}_{\theta }$, $S^{4}{}_{\theta }$ and $\sigma_{\theta }$ to reach a steady state. This effect of fluid viscosity is opposite to that of the hydraulic conductivity. Therefore, the lower the fluid viscosity, the faster fractures are initiated.

## Figures and Tables

**Table 2:** Parameters employed in the numerical calculation (FLAC3D).

Parameters	Value	Unit
Permeability, k	4.90219 × 10^−13^	m/(Pa·s)
Dry bulk modulus, K	118	MPa
Dry shear modulus, G	71	GPa
Biot’s coefficient, $\alpha_{B}$	1	
Porosity, n	0.4	
Water bulk modulus, $K_{f}$	2	GPa
Biot’s modulus, M	5	GPa
Wellbore radius, $r_{w}$	0.1	m
Outer radius, $r_{e}$	3	m
Initial pore pressure, $p_{0}$	2	MPa
Injection pressure, $p_{w}$	5	MPa

**Table 3:** Parameters employed in comparison with the numerical results (Sun et al., 2018) [44].

Parameters	Value	Unit
Wellbore radius, $r_{w}$	0.114	m
Influence radius, $r_{e}$	5	m
Permeability, k	1 × 10^−14^	m^2^
Porosity, n	0.4	
Dynamic viscosity of fluid, μ	0.005	Pa·s
Compression coefficient, β	1 × 10^−8^	Pa^−1^
Initial pressure, $p_{0}$	14.508	MPa
Injection pressure, $p_{w}$	14.771	MPa

**Table 4:** Parameters employed in comparison with the model (Zhou et al., 2021) [36].

Parameters	Value	Unit
Wellbore radius, $r_{w}$	0.1	m
Outer radius of seepage, $r_{e}$	3	m
Conductivity coefficient, $k_{w}$	1 × 10^−7^	m/s
Unit storage, $s_{r}$	1 × 10^−3^	m^−1^
Poisson’s ratio, ν	0.22	
Biot’s coefficient, $\alpha_{B}$	0.9	
Maximum horizontal stress, $\sigma_{H}$	20	MPa
Minimum horizontal stress, $\sigma_{h}$	15	MPa
Initial pore pressure, $p_{0}$	5	MPa
Injection pressure, $p_{w}$	15	MPa

**Table 5:** Comparison between experimental (Wang et al., 2017) [46] and predicted results.

Shale #	Tri-Axial Stresses x:y:z (MPa)	$\sigma_{t}$ (MPa)	*P_b_* from Equation (44) (MPa)	*P_b_* From This Paper (MPa)	Measured *P_b_* exp. (MPa)	Deviation (Equation (2)) %	Deviation (This Paper) %
1	11.06:14.49:17.89	0	12.57	9.08	8.96	−28.7	−1.28
2	7.35:10.93:14.44	0	7.48	5.40	6.58	−12.0	21.8
3	8.42:14.36:20.68	0	7.33	5.29	5.55	−24.3	4.79

Note: Biot’s constant α = 0.9, Poisson’s ratio ν = 0.3.

## Data Availability

Not applicable.
